# Hepatitis B and C viruses and survival from hepatocellular carcinoma in the Arkhangelsk region: a Russian registry-based study

**DOI:** 10.3402/ijch.v72i0.20282

**Published:** 2013-05-13

**Authors:** Maria Makarova, Alexandra Krettek, Mikhail Y. Valkov, Andrej M. Grjibovski

**Affiliations:** 1 International School of Public Health, Northern State Medical University, Arkhangelsk, Russia; 2 Nordic School of Public Health NHV, Gothenburg, Sweden; 3 Department of Internal Medicine and Clinical Nutrition, Institute of Medicine, Sahlgrenska Academy at University of Gothenburg, Gothenburg, Sweden; 4 Department of Radiology and Clinical Oncology, Northern State Medical University, Arkhangelsk, Russia; 5 Department of International Public Health, Norwegian Institute of Public Health, Oslo, Norway; 6 Institute of Community Medicine, University of Tromsø, Tromsø, Norway

**Keywords:** hepatocellular carcinoma, hepatitis B, hepatitis C, survival, Northwest Russia

## Abstract

**Introduction:**

Hepatocellular carcinoma (HCC) is one of the most common cancers worldwide. The prevalence of hepatitis B (HBV) and C (HCV) in Russia was 7.6 and 5.4 per 100,000, respectively. The aim of this study was to assess the proportion of HCV and HBV infection among HCC patients, to evaluate associations between HCV, HBV and stage of HCC and to compare survival of HCC patients by their HBV/HCV status in the Arkhangelsk region of northwest Russia.

**Materials and methods:**

A retrospective cohort study was conducted using data on all histologically confirmed HCC cases. Proportions of infected and non-infected HCC cases were calculated by Wilson's method. The associations between HBV, HCV and severity of HCC were assessed by Pearson's Chi-squared test. Survival data were presented using Kaplan–Meier curves and median survival. Survival time between the groups was compared using log-rank tests. Adjustment for potential confounders (sex, age groups, stage of HCC and cirrhosis stage by Child-Paquet scale) was performed using Cox regression.

**Results:**

There were 583 histologically confirmed HCC cases. The viral status was registered in 311 of patients with pre-mortem diagnosis, where 124 or 39.9% (95% confidence interval (CI), 34.4–45.4) had HBV, 54 or 17.4% (95% CI, 13.5–21.9) had HCV and 16 or 5.1% (95% CI, 3.2–8.2) were infected with both HBV and HCV. The median survival rates of patients were 3 months (95% CI, 2.3–3.8), 3 months (95% CI, 2.0–3.9) and 1 month (95% CI, 0.0–0.6) for patients with HBV, HCV and HBV and HCV, respectively. For virus-free patients, it was 5 months (95% CI, 3.5–6.5), log-rank test=10.74, df=3, p=0.013. Crude Cox regression showed increased risk of death for HBV and HBV and HCV groups in comparison with virus-free patients, and not reaching the level of statistical significance for HCV. After adjustment, the hazard ratios (HRs) decreased to non-significant levels or even reversed, with only exception for the group of patients infected with both hepatitis viruses.

**Conclusions:**

We found that more than half of HCC patients were infected with HBV or HCV. The study did not reveal an association between viral status of HCC patients and stage of HCC. The viral hepatitis may have an impact on survival of HCC patients.

Hepatocellular carcinoma (HCC) is the fifth most common cancer among men and the eighth most common cancer among women worldwide representing 6% of all cancers in 2009 (1). Men are affected more often than women (2). Nearly 630,000 new HCC cases are registered every year worldwide and 54,000 of them occur in Europe (3). The overall prevalence of HCC in Central Europe varies between 1 and 2 per 100,000, whereas in the northern part of Europe, the prevalence of HCC is less than 1 per 100,000 (4). In Nordic countries, the prevalence of HBV and HCV infection does not exceed 2% (5). According to the European Association for the Study of Liver Diseases, the number of HCC cases in Europe doubled between 2005 and 2010 (5). Probably, the quality of HCC diagnosis was improved. There is an association between the growth of viral hepatitis among all age groups and increasing number of HCC cases.

Mortality from HCC was 3.4 per 100,000 in 2007 worldwide with 77% of deaths occurring in developing countries (6). In countries with low prevalence of HCC, such as Norway (0.7 per 100,000), Sweden (1.1 per 100,000) and Finland (0.9 per 100,000), mortality from HCC varies between 0.5% and 2% of all cancer deaths (5).

In 1994, the International Agency for Research on Cancer classified hepatitis B (HBV) and C (HCV) viruses as carcinogens of the first class (7). The proportion of HCC among all cancers steadily increases in parallel to the increase in the prevalence of chronic forms of HBV and HCV (8). The risk for developing HCC among HBV- and HCV-infected persons is higher than that among non-infected individuals, particularly among those with mixed infections (8). Nearly 400 million people worldwide have chronic HBV infection and 170 million have chronic HCV infection (9).

In Russia, the proportion of HCC is 4.8% of all cancers (10). According to the official statistics, the prevalence of HCC in Russia increased from 3.7 per 100,000 in 2003 to 7.1 per 100,000 in 2010. The mortality from HCC in Russia was 5.8 per 100,000, while the 1-year survival rate was 11.5% in 2007 (10).

The prevalence of HBV and HCV in Russia was reported to be 7.6 and 5.4 per 100,000, respectively, with about 7% of infected had mixed HBV and HCV infection in 2009 (10). In 2009, the prevalence of HBV and HCV in Russia was 7.6 and 5.4 per 100,000, respectively, with about 7% of infected patients exhibiting mixed HBV and HCV infection (10). In the Archangelsk region, the prevalence was 4.8 per 100,000, with the highest level in the Amurski and Bashkirski regions (14.7 and 11.2 per 100,000) (10).

Given considerable health variations across Russia, national data may not be applicable to the northwestern part of the country. Indeed, information about HCC and its associations with HBV and HCV in northwest Russia is scarce, although the Arkhangelsk Regional Cancer Registry (ARCR) contains the data on all cancer cases providing a unique opportunity for epidemiological studies. The aim of this study is therefore to assess the proportion of HCV and HBV infection among HCC patients, evaluate associations between HCV, HBV and stage of HCC and to compare survival of HCC patients by their HBV and HCV status in the Arkhangelsk region of northwest Russia.

## Materials and methods

### Study subjects

The Arkhangelsk region is among the largest parts of the Euro-Arctic Barents region with a population of 1.27 million in 2010 (11). We conducted a retrospective cohort study using data on all histologically confirmed HCC cases registered in the ARCR from 2000 to 2008. Viral hepatitis B and C was diagnosed in the Regional Arkhangelsk Hospital. The diagnosis was based on the clinical and laboratory data (blood samples testing for Hbs or Hbc antigen). All physicians working in the Arkhangelsk region should regularly report on newly diagnosed cases of cancer to Arkhangelsk Regional Oncological Dispensary (AROD) using a standardized form. From this, the information is manually transferred to the ARCR. Detailed information about data collection routines and the information available from the ARCR have been presented elsewhere (12).

In the Arkhangelsk region, there is no universal registry for infectious diseases. Data on HBV and HCV in HCC patients were obtained from medical records at the AROD. We compared the list of HCC patients with archive data of the Region Hospital of the Arkhangelsk region as data from all infected patients were collected there. Our data were based on the medical cards, case history and treatment protocols.

Data on date of birth, date of HCC diagnosis, date of death, age, sex, stage of HCC according to the ICD-10 and cirrhosis stage by Child-Paquet scale were obtained from the ARCR.

Study subjects were grouped into 4 groups based on age: <60 years, 60–69 years, 70–79 years, 80 years and above. The HCC patients were grouped based on stage using the WHO recommendations (13). Stage I means a solid tumour less than 2 cm in diameter, stage II is a solid tumour exceeding 2 cm in diameter; stage III includes several forms: IIIA includes solid tumour in the liver less than 2 cm in diameter with invasive growth into the vessels or multiple liver tumours, while IIIB includes metastases of the lymphatic nodes. In this study, stages IIIA and IIIB were used as one group; stage IV is HCC with distant metastases (13).

The severity of cirrhosis was evaluated using the Child-Paquet scale: Child A (mild changes in the liver tissue), Child B (moderate changes) or Child C (severe changes) (14). This classification is based on the clinical indicators: the levels of blood bilirubin, albumin, prothrombin index, encephalopathy and ascite.

### Statistical analyses

Proportions of HCC cases with and without HBV and HCV were presented with 95% confidence intervals (CI) calculated using Wilson's method. The associations between HBV, HCV and severity of HCC were assessed by Pearson's Chi-squared test. Survival data were presented using Kaplan–Meier curves and median survival with 95% CI. Survival time between the groups was compared using log-rank tests. Adjustment for potential confounders (sex, age groups, stage of HCC and cirrhosis stage by Child-Paquet scale) was performed using Cox regression. Hazard ratios (HRs) were presented with 95% CI. All analyses were performed using SPSS software, version 15.0 (SPSS Inc., Chicago, IL, USA).

The study was approved by the Ethical Committee of the Northern State Medical University, Arkhangelsk, Russia on 21 October 2010, protocol number 21/10.

## Results

**Fig. 1:**
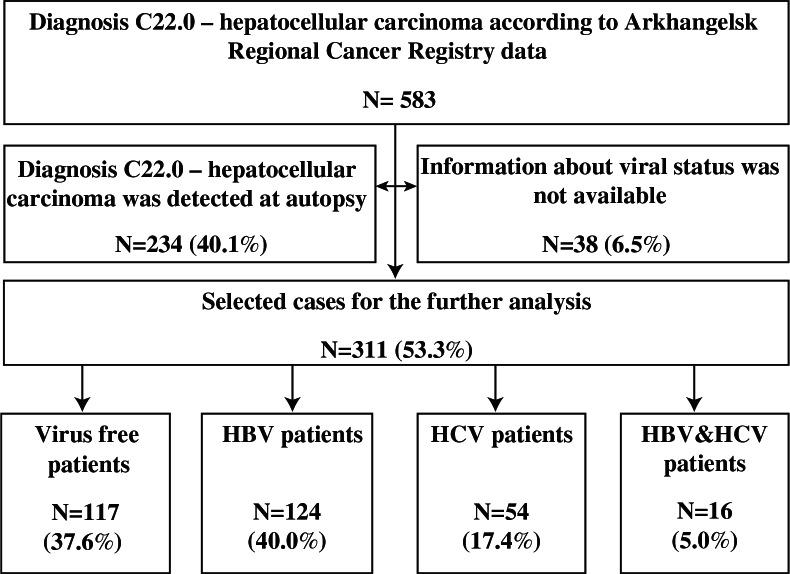
Sample description.

There were 583 histologically confirmed HCC cases during 2000–2008. Among them, 211 (36.2%), 92 (15.8%) and 28 (4.8%) patients had HBV, HCV infection or both forms of viruses, respectively. A total of 214 (36.7%) patients were virus-free. For 38 (6.5%) patients, information about viral status was not available. A total of 234 patients (40.1%) were diagnosed at autopsy. Further analysis of the data was performed for those where HCC was diagnosed pre-mortem. The flow chart is shown in Figure 1.

Viral status was registered in 311patients with pre-mortem diagnosis of viral hepatitis B and C. Among them, 124 or 39.9% (95% CI: 34.4–45.4) had HBV, 54 or 17.4% (95% CI: 13.5–21.9) had HCV and 16 or 5.1% (95% CI: 3.2–8.2) were infected with both HBV and HCV. Thus, the overall proportions of infected and non-infected patients were 62.3% (95% CI: 56–9–67.6) and 37.6% (95% CI: 32.4–43.2), respectively.

There were 128 (41.1%) females and 183 (58.9%) males. The mean age was 70.9 years for women and 71.3 years for men.

The proportion of patients with and without infection by basic demographic and clinical characteristics is shown in Table I. The cirrhosis stage among patients with HCC was significantly associated with viral status.

**Table I:** Socio-demographic and basic clinical characteristics of HCC patients in the Arkhangelsk region in 2000–2008 by their viral status

	Proportion of virus-free patients, % (n = 117)	Proportion of patients with viral hepatitis, % (n = 194)			
		
Characteristics		HBV (n = 124)	HCV (n = 54)	HBV and HCV (n = 16)	p*
Gender					
Male	73 (62.4%)	65 (52.4%)	35 (64.8%)	8 (50.0%)	0.266
Female	44 (37.6%)	59 (47.6%)	19 (35.2%)	8 (50.0%)	
Age (years)					
< 60	25 (21.4%)	20 (16.1%)	5 (9.3%)	5 (31.3%)	0.491
60–69	17 (14.5%)	21 (16.9%)	9 (16.7%)	3 (18.8%)	
70–79	39 (33.3%)	43 (34.7%)	24 (44.4%)	6 (37.5%)	
80 and above	36 (30.8%)	40 (32.3%)	16 (29.6%)	2 (12.5%)	
HCC stage					
Data not available	11 (9.4%)	22 (17.7%)	8 (14.8%)	5 (31.3%)	
I	15 (12.8%)	8 (6.5%)	5 (9.3%)	1 (6.3%)	0.117
II	16 (13.7%)	10 (8.1%)	2 (3.7%)	0 (0.0%)	
III	34 (29.1%)	34 (27.4%)	14 (25.9%)	2 (12.5%)	
IV	41 (35.0%)	50 (40.3%)	25 (26.3%)	8 (50.0%)	
Cirrhosis stage					
No cirrhosis	65 (55.6%)	45 (36.3%)	33 (61.1%)	7 (43.8%)	<0.0001
Child A	37 (31.6%)	35 (28.2%)	5 (9.3%)	4 (25.0%)	
Child B	14 (12.0%)	26 (21.0%)	9 (16.7%)	3 (18.8%)	
Child C	1 (0.9%)	18 (14.5%)	7 (13.0%)	2 (12.5%)	

* Calculated using Pearson's Chi-squared test.

**Fig. 2:**
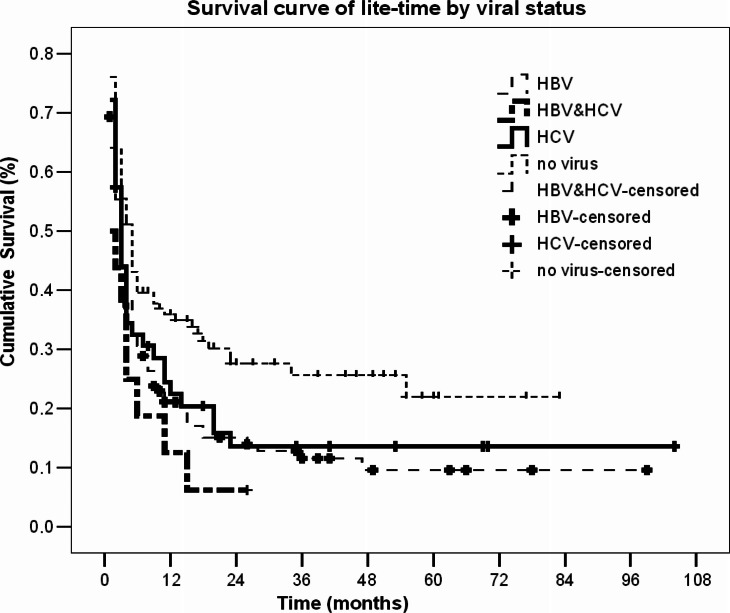
Survival of HCC patients in the Arkhangelsk region in 2000–2008 by their viral status. Data show that non-infected HCC patients have more survival time compared with patients infected with viral hepatitis B and C virus.

Survival curves by viral status are shown in Figure 2.

The median survival of patients was 3 months (95% CI, 2.3–3.8), 3 months (95% CI, 2.0–3.9) and 1 month (95% CI, 0.0–0.6) for patients with HBV, HCV and HBV and HCV, respectively, while virus-free patients had a median survival of 5 months (95% CI, 3.5–6.5), log-rank test=10.74, df=3, p=0.013.

Crude Cox regression showed an increased risk of death for all virus groups in comparison with virus-free patients, although not reaching the level of statistical significance for HCV. However, after adjustment for potential confounders, the HR decreased to non-significant levels or even reversed, with the only exception for the group of patients infected with both hepatitis viruses (Table II).

**Table II:** Crude and adjusted hazard ratio for survival of HCC patients in the Arkhangelsk region in 2000–2008 by their viral status

		95% CI			95% CI	
				
Factors	HR crude	Lower	Upper	HR adjusted^a^	Lower	Upper
Viral status						
Virus-free	1.00	Reference	Reference	1.00	Reference	Reference
HBV-infected	1.43	1.07	1.91	1.13	0.83	1.53
HCV-infected	1.34	0.93	1.93	1.04	0.71	1.52
HBV- and HCV-infected	1.89	1.09	3.27	2.10	1.16	3.78

a Adjusted for sex, age groups, cirrhosis stage and stage of HCC.

## Discussion

This study is one of the first to investigate the association of viral hepatitis status and its impact on HCC not only in northwest Russia but also in the Russia Federation in general. We found that more than half of HCC patients in the Arkhangelsk region were infected with viral hepatitis B and C. No association was determined between the viral status of HCC patients and the stage of HCC. However, viral hepatitis B and C had an impact on survival of HCC patients.

It has been established that viral hepatitis B and C activate metabolism and biosynthesis of lipids, which damages hepatic tissue. Viral infection includes lipogenic gene expression within hepatic cells that promote carcinogenesis (15). The proportion of HCC patients infected by HBV and HCV in this study constitutes 39.9 and 17.4%, respectively. Earlier regional registry-based studies report that approximately 40–55% of HCC patients have HBV in anamnesis (16) and that the proportion of HCV-infected individuals is 25–30% (17). Furthermore, official statistics shows that more than 50% of HCC patients in Russia are infected with either HBV or HCV (10, 17). The typical habitus of a viral-infected patient is men of 52–59 years, alcohol-abused, smokers, with specialized secondary education.

The only factor that had uneven distribution among HCC patients in our study was cirrhosis frequency. Cirrhosis stage as evaluated by Child-Paquet scale was based on the calculations of the laboratory tests of the blood samples, which were unavailable for non-infected HCC patients. Our study demonstrated that 56.2% of infected HCC patients had liver lesions due to liver cirrhosis. We found a large proportion of advanced forms of liver cirrhosis in virus-infected patients. Child B and C were 35.5% for HBV, 29.7% for HCV and 31.3% for HBV and HCV, respectively, while this proportion in non-infected patients was 12.9%. The cirrhosis is not an obligatory stage for HCC. Probably, there was underestimation of the cirrhosis condition. In the Arkhangelsk region, the detection of fibre tissue in hepar begun in 2011. The liver cirrhosis among infected is probably a pre-stage in the development of HCC (8, 18).

Since our study is based on the registry data and information about alcohol consumption, another toxic factor was not available for the analysis. It might be supported that cirrhosis was a background condition of these patients, which was not diagnosed.

We performed subanalysis of survival for this group of patients. There were 19 cases of death from 61 non-infected patients with cirrhosis stage 1–2. Thus, 19 cases of death were confirmed at autopsy. The cause of death was not HCC. The causes of deaths among these 19 cases were road accidents (3 cases), cardiac infarction (9 cases), stroke (4 cases) and suicides (3 cases). Also, within the frames of diagnosis C22 (HCC), metastatic liver cancer could be included. This fact supported the initially large number of deaths. But there were 11 patients who lived 2–19 months after the diagnosis was established. The problem of diagnosing HCC influences the oncological statistics. In the Arkhangelsk region, there were several misdiagnosed HCC cases. The clinicians diagnosed metastatic liver cancer as C22, and such cases were included into the registry. During autopsy the diagnosis was changed, but these new data were not registered. The official statistics is published 2–3 months before the reports of pathologists become available leading to some differences between clinical diagnosis and the diagnosis made by a pathologist. In our study, there was no association between viral status and HCC stage. Stages III and IV prevailed both among virus-free (29.1 and 35.0%, respectively) and infected patients (25.8 and 42.8%, respectively). The studied population was comparable to the HCC population as of Russia, and for the European countries. The probable explanation is that infected patients are thoroughly followed up after diagnosis of HBV and/or HCV having annual radiological examination of the liver. In contrast, virus-free patients likely have less aggressive tumours are examined less frequently. It may lead to the development off the HCC advanced stages. On the contrary, Wörns et al. demonstrated an association between viral status and HCC stage (19). According to their data, virus-free patients have I or II HCC stages (32.6 and 42.8%, respectively). The results by Sylla et al. also indicate that more than 60% of infected patients exhibit HCC stages III or IV stages, while among virus-free patients stage I and II predominate (46.5 and 31.6%, respectively) (20). The reason is that viral hepatitis impairs liver cells. Therefore, HCC develops on the formed pathological substrate of damaged liver tissue. While virus-free patients have no pathological changes within liver tissue, stages I and II prevail. The data about duration of viral hepatitis were not available. Patients infected with viral hepatitis B or C had annual medical examinations, including biochemical blood analysis and ultrasound hepar examination. Virus-free patients without evident cancer signs could undergo a prophylactic medical examination, or visit the doctor if concerned (e.g. family medical history), or, as mentioned above, within routine check-ups. Furthermore, we have investigated the impact of viral infection on patient survival. In this study, Kaplan–Meier estimates initially suggested that viral status might shorten survival of patients with HCC. The proportion of patients who survived was 2 times higher among virus-free patients by the 5th year (22.4% for virus-free vs. 9.5% for HBV; 13.0% for HCV; 6.1% for HBV and HCV). However, after adjustment for potential confounders (sex, age groups, cirrhosis stage, stage of HCC, data of viral hepatitis diagnostics), the survival of both HBV and HCV infected HCC patients was similar to the survival of non-infected patients. Only a small group of 16 HCC patients having mixed infection of HBV and HCV revealed significantly poorer prognosis as compared to non-infected in the adjusted model. This is consistent with the evidence that viral hepatitis worsens the survival of patients with HCC. In contrast to our data, some authors have shown that HCC patients with HBV or HCC monoinfection exhibit survival time up to 3 times shorter compared to non-infected patients (8, 15). The adjustment allows us to get true data in the researched population. We could not show this due to the small sample size of this study—our analysis included only 311 selected patients. Otherwise, our sample includes all HCC cases in the Arkhangelsk region.

This retrospective cohort study has several limitations as it only allows assessing existing data. However, it is useful for exploring an association between survival of patients with HCC and viral hepatitis status. HCC is a rare disease. Moreover, during our study more than 40% of HCC cases were excluded as cases were diagnosed at autopsy. Vaktskjold et al. report that ARCR contains quality data providing estimations of the cancer incidence in the Arkhangelsk region. The authors also consider that the data from ARCR may show the true cancer situation in the Arkhangelsk region (12). ARCR covers all cases of HCC.

During this study we revealed that the ARCR was developed for medical purposes. It does not contain enough specific information for our research aims, including information about viral status. Additionally, there is no common database for viral hepatitis in the Arkhangelsk region. Data about such patients are available only based on medical records and case histories. The searching of the listed data (hepatitis status) results in the data about viral status of 6.5% HCC patients being missing. It was technically impossible to assess the influence of potential confounders that are associated with the outcome of the disease (alcohol consumption, diabetes status, etc.) (21). Also, we did not assess the association between type of treatment of viral hepatitis and the outcome of HCC.

The strength of the study is that it is registry-based, which includes all cases of HCC for the period 2000–2008 in the Arkhangelsk region. The ARCR covers all geographic areas of the Arkhangelsk region. Therefore, ARCR should reflect the whole situation of HCC in the Arkhangelsk region.

Oncological diseases play a leading role among causes of death and invalidity of the population. Understanding the causes allow us to reduce the morbidity and mortality of cancer. Many studies have demonstrated that HCC correlates with viral hepatitis. However, these studies evaluating the association of HCC and viral infection were not conducted in most regions of Russia. Moreover, during our study we revealed another problem – misdiagnosis of liver cancer and underestimation of viral hepatitis B and C. This reflects the quality of medical services for the population, and, as a result, the quality of public health of the Arkhangelsk region.

## Conclusions

We have found that more than half of the HCC patients were infected with HBV or HCV. The study did not reveal an association between the viral status of HCC patients and the stage of HCC. The viral hepatitis may have an impact on survival of HCC patients.
